# Photodynamic Inactivation of *Pseudomonas aeruginosa* by PHEMA Films Loaded with Rose Bengal: Potentiation Effect of Potassium Iodide

**DOI:** 10.3390/polym13142227

**Published:** 2021-07-06

**Authors:** Ana M. López-Fernández, Ignacio Muñoz Resta, Rosa de Llanos, Francisco Galindo

**Affiliations:** 1 Departamento de Química Inorgánica y Orgánica, Universitat Jaume I, Av. V. Sos Baynat s/n, 12071 Castellón, Spain; lopezan@uji.es (A.M.L.-F.); resta@uji.es (I.M.R.); 2 Unidad Predepartamental de Medicina, Universitat Jaume I, Av. V. Sos Baynat s/n, 12071 Castellón, Spain

**Keywords:** PHEMA, Rose Bengal, photodynamic inactivation, antimicrobial materials, bactericidal materials, *Pseudomonas aeruginosa*, singlet oxygen, photosensitizers, light

## Abstract

Four formulations have been used to produce different poly(2-hydroxyethyl methacrylate) (PHEMA) thin films, containing singlet oxygen photosensitizer Rose Bengal (**RB**). The polymers have been characterized employing Thermogravimetric Analysis (TGA), Attenuated Total Reflectance Fourier Transform Infrared Spectroscopy (ATR-FTIR) and UV-vis Absorption Spectroscopy. When irradiated with white light (400–700 nm) films generated singlet oxygen (^1^O_2_), as demonstrated by the reactivity with ^1^O_2_ trap 9,10-dimethylanthracene (**DMA**). Material with the highest **RB** loading (polymer **A4**, 835 nmol **RB**/g polymer) was able to perform up to ten cycles of **DMA** oxygenation reactions at high conversion rates (ca. 90%). Polymer **A4** was also able to produce the complete eradication of a *Pseudomonas aeruginosa* planktonic suspension of 8 log_10_ CFU/mL, when irradiated with white light (total dose 72 J/cm^2^). The antimicrobial photodynamic effect was remarkably enhanced by adding potassium iodide (100 mM). In such conditions the complete bacterial reduction occurred with a total light dose of 24 J/cm^2^. Triiodide anion (I_3_^−^) generation was confirmed by UV-vis absorption spectroscopy. This species was detected inside the PHEMA films after irradiation and at concentrations ca. 1 M. The generation of this species and its retention in the matrix imparts long-lasting bactericidal effects to the **RB**@PHEMA polymeric hydrogels. The polymers here described could find potential applications in the medical context, when optimized for their use in everyday objects, helping to prevent bacterial contagion by contact with surfaces.

## 1. Introduction

Infections by microorganisms cause the death of 13 million people worldwide annually [1], and the situation is worsening due to the emergence of antibiotic-resistant strains [2]. In hospital settings, the occurrence of nosocomial infections has become a public health problem, involving an economic burden of $33 billion each year in the United States [3]. Specifically, *Pseudomonas aeruginosa* is responsible for 10–15% of such hospital-acquired infections and is associated with high mortality rates (18–61%) [4]. Hence, it is not surprising that materials with antimicrobial properties are highly demanded by society. The majority of antimicrobial materials are designed using polymers containing groups with well-known microbiocidal properties, like ammonium and phosphonium cations [5,6,7,8,9,10,11,12,13]. In addition to its application in health, surface contamination is also an issue for the food industry and hence, solutions are actively sought in the Materials Science area [14]. An alternative strategy that is receiving increasing attention is the use of visible light to activate materials via the encapsulation of a photosensitizer capable of converting the absorbed energy into cytotoxic species. Historically, the development of this scientific discipline started with studies on photosensitization in solution, this approach being termed as antimicrobial photodynamic inactivation (aPDI), among other denominations [15,16]. In aPDI the combination of light and photoactive molecules gives rise to reactive oxygen species (ROS). To summarizing, the mechanism of photosensitization implies the absorption of light by a molecule that is activated to an excited state. From that state, electron or energy transfer can take place (type I and II mechanisms, respectively) to the surrounding ground state oxygen. This process leads to the formation of radical species (hydroxyl radical, superoxide anion, via type I mechanism) and/or singlet oxygen (^1^O_2_, via type II mechanism), which induce the killing of pathogenic microorganisms. The above process has often been described to occur for photosensitizers in solution [17,18,19,20,21,22,23], and also when the photosensitizers are immobilized on a polymeric matrix [24,25,26,27]. Polymeric matrix recently used in aPDI include, among others, polystyrene nanofibers [28], polylactic acid [29], polyolefin thermoplastic elastomers [30], pyridinium modified Merrifield resins [31], polyether block amide [32], chitosan [33], wool keratine [34], cellulose [35], nylon [36] and polyacrylonitrile [36].

One of the most popular polymers used in Biomedicine is poly(2-hydroxyethyl methacrylate) (PHEMA). Due to its excellent biocompatibility, PHEMA has been used for the manufacture of contact lenses [37], ureteral stents [38] and platforms for neural growth [39]. But the applications of PHEMA materials go beyond the medical field and PHEMA can be used in the development of oxygen sensing materials [40], ion-selective membranes [41], batteries [42], and polymers for environmental remediation [43]. Due to the transparency of such materials, the encapsulation of optically active systems is especially attractive. Since 2005 we have designed and synthesized tailored coloured or emissive PHEMA hydrogels for various sensing applications [44,45,46,47,48,49,50], and more recently we have encapsulated a photoactive hexanuclear molybdenum complex in a PHEMA matrix. It has been found to be active against *Staphylococcus aureus* after irradiation with visible light [51]. As a continuation of such work, we are now reporting the encapsulation of the photosensitizer Rose Bengal (**RB**) into a matrix of cross-linked cationic PHEMA (**RB**@PHEMA) displaying photo-antibacterial properties against Gram-negative bacteria *P. aeruginosa* in planktonic suspension. The PHEMA-related materials described above use swelling [52] and covalent binding [53] as strategies to incorporate photosensitizers. On the contrary, the strategy used here consisted of the dissolution of underivatized **RB** in the polymerizable formulation of monomers and crosslinker.

In the aPDI context, it has been described that iodide anion (I^−^) can boost the photodynamic killing of bacterial and fungal species [54,55,56,57,58,59,60,61,62,63,64]. This is especially appealing for anionic photosensitizers, like **RB**, which are particularly inefficient against Gram-negative bacteria in solution [57]. However, in combination with I^-^ the antimicrobial photodynamic action is enhanced several orders of magnitude.

In the present study, the photo-antimicrobial properties of **RB**@PHEMA films have been demonstrated against *P. aeruginosa.* In addition, this is the first time that a photoactive PHEMA material is used in combination with potassium iodide, which has remarkably enhanced its bactericidal activity. 

## 2. Materials and Methods

### 2.1. Materials

Rose Bengal disodium salt (**RB**, ≥85%, Sigma, St. Louis, MO, USA), 2-hydroxyethyl methacrylate (**HEMA**) (97%, Acros Organics, Waltham, MA, USA), (3-acrylamidopropyl)trimethyl-ammonium chloride solution (**ATAC**) (75 wt.% in H_2_O, Aldrich Chemistry, St. Louis, MO, USA), poly(ethylene glycol) dimethacrylate average Mn 550 (**PEGDMA**) (Aldrich Chemistry, St. Louis, MO, USA), α,α-azoisobutyronitrile (**AIBN**) (≥95%, Glentham, Corsham, UK), 9,10-dimethylanthracene (**DMA**) (97%, Alfa Aesar, Waltham, MA, USA), potassium iodide (analytical grade, Scharlau, Barcelona, Spain) were used as received. The solvents were spectroscopic grade (Scharlab, Barcelona, Spain).

### 2.2. Hydrogel Synthesis

The polymeric hydrogels were synthesized according to the following procedure [51]: Rose Bengal disodium salt (from 0.005 to 0.1% wt., relative to the total mass of monomers and crosslinker) was dissolved in a mixture of **HEMA**:**ATAC**:**PEGDMA** (85:5:10 wt.%, respectively). Then, **AIBN** was added and dissolved (1 wt.% relative to the total mass of monomers and crosslinker). A small part of this solution was pipetted into the narrow space formed between two microscope glass slides separated by two thin coverslips at the edges. The system was placed in an oven at 85 °C for 15 min. After polymerization, the two glass slides were separated and the film was removed and washed with distilled water to eliminate any unreacted material. To facilitate the unmoulding process, the glass slides were previously treated with silicone oil and put in an oven at 200 °C for 60 min. The films were typically 75 × 25 mm^2^ and could be cut into pieces to obtain samples with the desired weight.

### 2.3. Characterization

The samples were analysed by Attenuated Total Reflectance Fourier Transform Infrared Spectroscopy (ATR-FTIR) (Jasco, Tokyo, Japan) with a Jasco FT/IR 6200 type A with a TGS detector. The spectra were recorded in the range 4000–400 cm^−1^, with 128 scans per spectrum and a resolution of 4 cm^−1^. Thermogravimetric analyses (TGA) (Mettler Toledo, Columbus, OH, USA) were performed with a TG-STDA Mettler Toledo model TGA/SDTA851e/LF/1600 from 25 °C to 500 °C, at a heating rate of 10 °C/min under air atmosphere. All measurements were done in 40 µL platinum crucibles and an empty platinum crucible was used as a reference. Sample masses of ca. 10 mg were used. The thickness of the films was measured with a Mitutoyo Digimac Caliper (500 series) (Mitutoyo Ltd, Andover, UK). UV-vis spectra were recorded on a Cary 60 UV-vis spectrophotometer (Agilent Technologies, Santa Clara, CA, USA). The films were maintained between two microscope slides during the absorption measurements.

### 2.4. Photochemical Studies

Photooxygenation reactions were performed inside 10 mL vials containing 50 mg of the polymeric film and 3 mL of the ^1^O_2_ trap **DMA** (10^−4^ M in EtOH:H_2_O, 1:1, v:v). Irradiations were carried out using two white light LED lamps (9 W each, ca. 400−700 nm emission output; see Figure S1 in the Electronic Supporting Information) placed 2.5 cm away from the vial under continuous stirring (light irradiance 180 mW/cm^2^ for each lamp at 2.5 cm). The evolution of the photoreactions was monitored over time by means of UV−vis absorption spectrophotometry (decrease of absorbance at 377 nm). The kinetic traces were fitted to the following pseudo-first order model [51]: ln (A/A_0_) = −k_obs_ · t, where A_0_ is the initial absorbance of **DMA**, A is the absorbance of **DMA** at time t, and k_obs_ is the observed kinetic constant.

### 2.5. Microorganisms and Growth Conditions

The Gram (−) bacterial strain *P. aeruginosa* ATCC 27.853 was acquired from the American Type Culture Collection (ATCC, Rockville, MD, USA). Microorganisms were routinely seeded on Mueller Hilton Agar (MHA) (Scharlau, Spain) and cultured aerobically overnight at 35 °C.

### 2.6. Antimicrobial Photodynamic Inactivation Experiments

The aPDI experiments were carried out by exposing a sample of bacteria in planktonic state (see below for details) to 25 mg films loaded with/without **RB** and with/without 100 mM KI solution. The following samples and controls were performed: cells without KI (A, control); cells with KI (B, control); cells without KI and incubated with **RB**-films (C, sample); cells without KI and incubated with no **RB**-films (D, control); cells with KI and incubated with **RB**-films (E, sample); cells with KI and incubated with no **RB**-films (F, control). These six groups were subjected to irradiation (named with uppercase letters, A-F) and in parallel, the same groups were kept in darkness as controls (named with lowercase letters, a–f). Irradiations were carried out with a source of white light LED TENKO ECO 50 W (output 400–700 nm, light irradiance 80 mW/cm^2^ at a distance of 2.5 cm; see Figure S2 in the Electronic Supporting Information). Previously to the aPDI experiments, films loaded with/without **RB** were first sterilized by dipping them in 70% ethanol and subsequently air-dried.

The bacterial inoculum was prepared in sterilised distilled water and adjusted to 1.00 ± 0.03 on the McFarland scale (microbial suspensions containing >3 × 10^8^ bacteria/mL). An aliquot of 1 mL of the bacterial suspension was equally distributed to a 12-well plate, and then, either 1 mL of sterilized distilled water or 1 mL of 200 mM KI stock solution was also transferred to the plate to achieve a final microbial suspension of ≈10^8^ bacteria/mL and a final 100 mM KI concentration. Irradiation of the 12-well plate containing samples and controls (A–F) were exposed to white light keeping 2.5 cm of distance between the lamp and the plates, at room temperature and under agitation in an orbital shaker (120 rpm). Aliquots of 75 µL of samples and controls were collected at different times of light exposure (5 min: 24 J/cm^2^; 10 min: 48 J/cm^2^; 15 min: 72 J/cm^2^ and 30 min: 144 J/cm^2^). For the darkness condition, the 12-well plate containing samples and controls (a-f) were kept in the dark for 30 min at room temperature and under agitation in an orbital shaker (120 rpm). Aliquots of 75 µL of samples and controls were collected at 30 min. 

After completing the aPDI protocol (or dark incubation), relative cell survival was evaluated for each sample and control aliquots from both irradiated and dark conditions, by counting colony-forming units (CFU) on MHA. To do this, the initial suspensions (75 µL aliquots of samples and controls) were serially diluted 10-fold in sterile distilled water to give concentrations of 10^−1^ to 10^−5^ times. Drops of 5 µL of each dilution and the original suspension were spotted onto MHA plates (plate-serial dilution spotting) using a multichannel pipette, and were then incubated overnight at 35 °C. After 24 h, at least one dilution level yielded 15–60 CFU was counted, and CFU/mL was calculated. 

### 2.7. Statistical Analysis

All the experiments were performed in three or four independent replicates. Results are expressed as mean ± standard deviation. Mean comparisons were performed after verifying homogeneity of variances using Barlett’s test. In cases of homoscedasticity, differences among treatments were analysed by one-way analysis of variance (ANOVA) followed by Tukey’s multiple comparison test. If heterogeneity of variances existed, robust tests were carried out as follows. Welch’s test was used to check for differences among treatments, with Game-Howells’s test used to establish differences among treatments. Comparisons of the means with *p* values less or equal than 0.05 were regarded as significantly different in all tests. All statistical analysis was performed using the IBM SPSS Statistics, version 27 (SPSS Inc., Chicago, IL, USA).

## 3. Results and Discussion

### 3.1. Preparation and Characterization of RB@PHEMA Films

**Figure 1:**
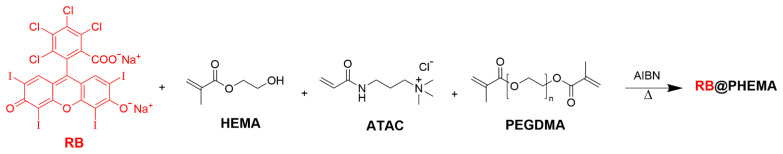
Structures of photosensitizer **RB** and monomers (**HEMA**, **ATAC**) and crosslinker (**PEGDMA**) used to synthesize **RB**@PHEMA.

Synthesis of PHEMA films was carried out by thermally initiated radical polymerisation (**AIBN** as initiator) as previously reported [51]. The corresponding amount of **RB** disodium salt (from 0.005 to 0.1% wt.) and initiator (1% wt.) was dissolved in a mixture of monomers 2-hydroxyethyl methacrylate (**HEMA**)/(3-acrylamidopropyl)trimethyl-ammonium chloride solution (**ATAC**) and cross-linker poly(ethylene glycol) dimethacrylate (**PEGDMA**, average Mn 550). The indicated % wt. of **RB** and **AIBN** are relative to the total amount of monomers and crosslinker. Formulas of **HEMA**, **ATAC** and **PEGDMA** can be seen in Figure 1.

**Figure 2:**
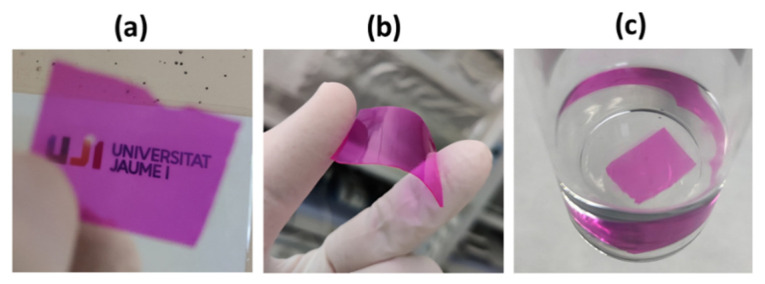
(**a**,**b**): pictures of a representative film of polymer **A4** (size 30 × 25 mm^2^) illustrating its transparency and flexibility; (**c**): picture of another film of polymer **A4** (size 14 × 10 mm^2^) immersed in water for four months, illustrating the absence of **RB** leaching.

The chosen internal proportion of the acrylic polymerizable molecules **HEMA**:**ATAC**:**PEGDMA** was 85:5:10 (in wt.%). Such formulation was chosen considering our previous experience with encapsulation of optically active molecules [44,45,46,47,48,49,50,51]. In previous investigations, it was found that the appropriate choice of monomers and cross-linkers gave rise to materials with no detectable dye leaching outside the polymeric matrix, although this feature depends largely on the nature of the dye. For instance, a very hydrophobic sensing dye like 1,2-diaminoantraquinone remained trapped in the interior of PHEMA cross-linked films for months [46,47,50]. However, for anionic xanthenic dyes, the use of a cationic monomer is mandatory to create the appropriate electrostatic attraction as previously demonstrated for PHEMA entrapping the anionic sensor **DAF-FM** [49]. In this regard, **ATAC** monomer, with a permanent positive charge, was used for this purpose, since it allows the formation of an ion pair with the negatively charged **RB** (without the need of covalent attachment). Regarding the crosslinker, it was previously found that **PEGDMA** in a low proportion (10%) was appropriate to produce films with good flexibility, strength, manipulability and the desired permeability to small chemical species in aqueous solutions. Polymerization was carried out inside a mould made with two glass slides and two coverslips, leading to thin films of up to 75 × 25 mm^2^ (width of 180 ± 10 µm measured with a digital micrometre), which were easily cut into small pieces using scissors (leading to small rectangular pieces of 50 mg or 25 mg for irradiations). Pictures of representative examples of **RB**@PHEMA were taken to illustrate their transparency (Figure 2a) and flexibility (Figure 2b). An advantageous feature of the herein proposed encapsulation method in comparison to others previously reported, stems from the fact that no derivative of **RB** bearing a polymerizable group is required for the entrapment of photosensitizers in polymeric matrices [53]. Another difference of the followed approach is that the amount of entrapped **RB** can be precisely controlled just by dissolving the desired concentration of **RB** in the polymerizable mixture (see discussion below regarding UV-vis measurements of encapsulated **RB**). The alternative methodology implying dye loading by swelling of the matrix is also a frequently used strategy [52] but, in such cases, it is difficult to control the concentration of dye entrapped into the polymer. However, for the method described here, the final concentration of the photosensitizer inside the matrix is exactly the initial concentration before polymerization. As mentioned in the Introduction, we have successfully used this strategy for entrapping a variety of optically active molecules, from aromatic ketones [46,47,50] and xanthenic dyes [49], to flavylium cations [44,45] and molybdenum clusters [51], all retaining their properties after the thermal polymer formation.

Several concentrations of **RB** were assayed to find the one yielding optimal photochemical activity. Thus, four films (**A1**–**A4**) were prepared with the above-mentioned formulation of **HEMA**, **ATAC** and **PEGDMA** but differing only in RB loading, ranging from 0.005% wt. to 0.1% wt. (or from 42 to 835 nmol RB/g polymer). An additional fifth film (**B**) was also prepared, without the photosensitizer, and used as a control. The concentration of **RB** in the films can be seen in Table 1.

**Figure 3:**
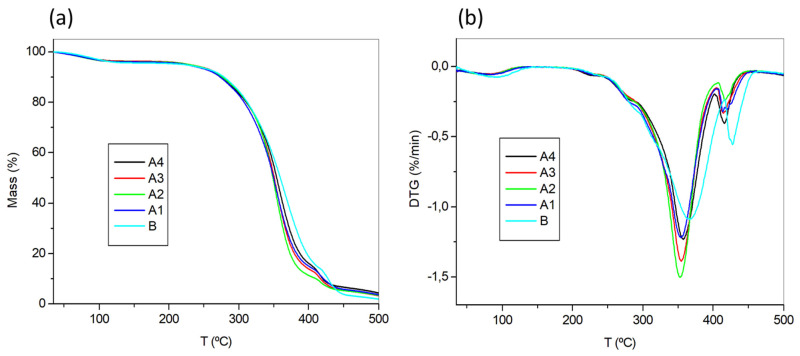
(**a**) Thermograms for films **A1**–**A4, B** and (**b**) the first derivative of such thermograms.

The thermal properties of the films were determined by thermogravimetric analysis (TGA). As can be seen in Figure 3, for the thermograms and in Table 1 for the data, all the polymers under study displayed onset degradation temperatures (*T*_5%_) around 225 °C and maximum degradation temperatures (*T*_max_) around 356 °C. These values are comparable to similar PHEMA materials described in the literature, with differences attributable to variations in the monomer/cross-linker composition and the polymerization method. As illustrative references, D. Park et al. reported a polymer made with **HEMA** and ethylene glycol dimethacrylate (**EGDMA**) in a ratio of 95:5 displaying a *T*_5%_ of ca. 253 °C and a *T*_max_ of ca. 350 °C [65]. More recently, Muñoz-Bonilla et al. reported an antimicrobial material made with **HEMA** and poly(ethylene glycol) diacrylate (**EGDA**) in a ratio of 5:1, which have a *T*_5%_ of ca. 317 °C and a *T*_max_ of ca. 388 °C [66]. Polymer **B**, without **RB,** resulted slightly more stable than **A1–A4**, which have similar properties, irrespective of the photosensitizer concentration.

**Figure 4:**
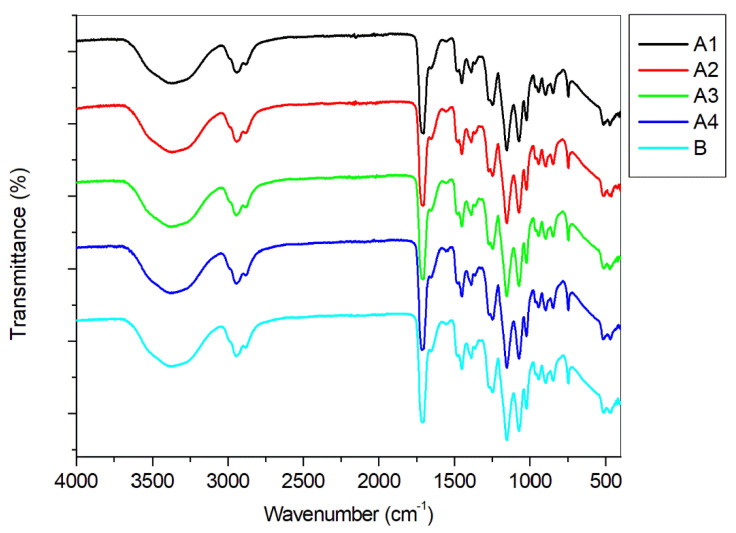
ATR-FTIR analysis of films **A1**–**A4**, **B**.

Analysis of the materials by ATR-FTIR yielded a series of spectra with almost identical features, as expected, since the concentration of photosensitizer is too low to be detected by this technique (Figure 4). The main bands of the spectra were the corresponding to hydroxyl stretching (around 3700–3100 cm^−1^), carbonyl stretching (around 1712 cm^−1^) and characteristic vibrations of C-O bond in the range 1300–1000 cm^−1^. Similar features were described for other PHEMA materials reported in the literature [66].

**Figure 5:**
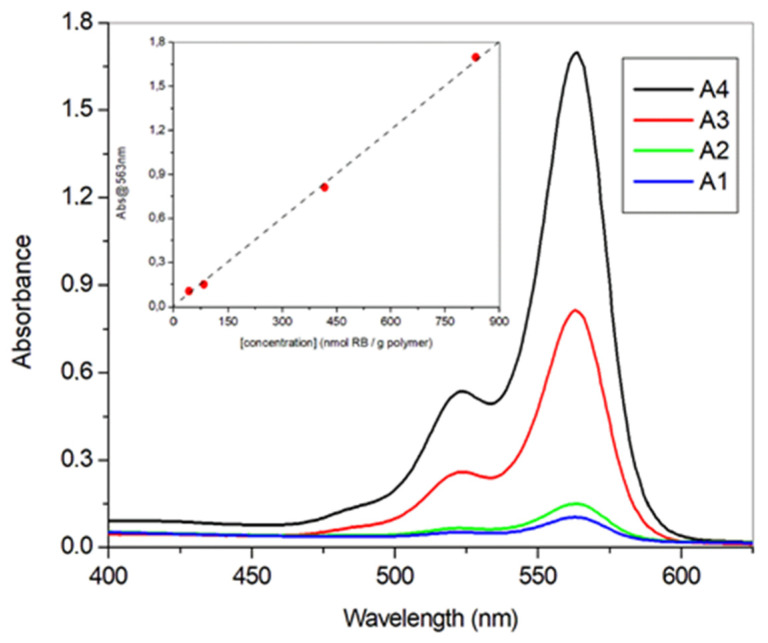
UV-vis absorption spectra of **A1**–**A4** films. Inset: absorbance at 563 nm as a function of **RB** loading (linear fit with R = 0.9995, slope = 0.00203 ± 0.00004).

The optical characterization of every film was carried out by UV-vis absorption spectroscopy, taking advantage that the materials were transparent enough to make reliable direct measurements. Thus, placing the films in the optical path of the spectrophotometer yielded the spectra shown in Figure 5. Comparison of absorbance at 563 nm vs. **RB** concentration follows a linear relationship, suggesting no important aggregation inside the films. Moreover, the shape of the spectra remains invariant irrespective of the concentration tested. This statement must be taken cautiously since spectroscopically undetectable aggregates can lead to excitation energy trapping and hence, energy waste, as recently demonstrated with Phloxine B by San Román and co-workers [67]. The same group has recently described the behavior of **RB** in PHEMA films from a photophysical standpoint, at concentrations of photosensitizer one or two orders of magnitude higher than ours (ca. 1 mM for film **A4**). These authors detected noticeable self-quenching effects above 10 mM [68].

Despite the existence of some experimental uncertainty on the determination of film thickness, and considering a concentration of **RB** inside the matrix of ca. 1 mM for **A4**, application of the Beer-Lambert law affords an extinction coefficient for this photosensitizer of ca. 10^5^ M^−1^ cm^−1^ inside the film, in agreement with the value described in the literature (105,000 M^−1^ cm^−1^ for the monomeric form in water) [69].

Films were soaked in water for several months to test the potential leaching of the photosensitizer out of the polymeric matrix. After four months immersed in water no **RB** was detected in solution as measured by UV-vis absorption spectroscopy (see a picture of A4 in Figure 2c). Likely, the presence of the **ATAC** monomer is responsible for maintaining the photosensitizer electrostatically bound to the polymeric matrix. A film containing **RB** (835 nmol/g polymer) made with only **HEMA**:**PEGDMA** (90:10) was prepared to test this hypothesis and, as expected, partial leaching of the photosensitizer took place after hours of immersion in water (data not shown).

### 3.2. Photochemical Generation of Singlet Oxygen by RB@PHEMA Films

**Figure 6:**
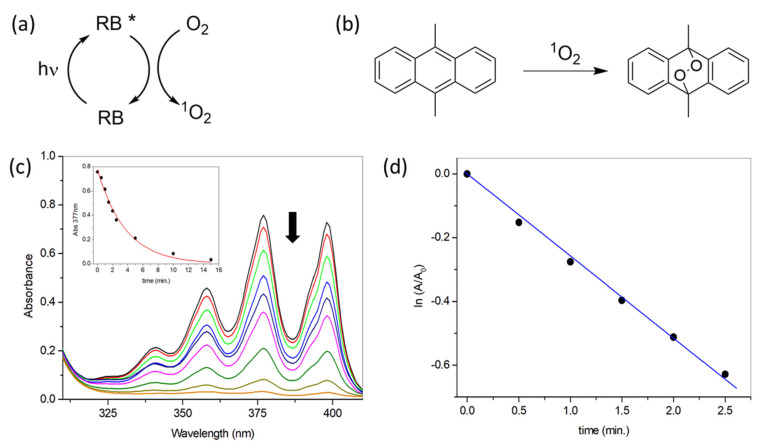
(**a**) Photosensitized generation of ^1^O_2_ from irradiated **RB**; (**b**) **DMA** reaction with ^1^O_2_; (**c**) Illustrative example of **DMA** reactivity monitored by UV-vis absorption spectroscopy; (**d**) Illustrative fitting to a pseudo-first order kinetic model, using selected irradiation as an example. Note that to obtain the values shown in Table 1, the procedure was performed by triplicate and the values averaged.

The ability of **A1**–**A4** films to generate singlet oxygen upon irradiation was tested using the well-known probe of this species 9,10-dimethylanthracene (**DMA**). This is a chemical trap of ^1^O_2_ frequently used to estimate the photoactivity of molecules and polymers. **DMA** absorption at 300–400 nm fades out upon reaction with ^1^O_2_ due to the formation of the corresponding endoperoxide, not absorbing in such spectral region. In Figure 6a it is shown the fundamentals of photosensitized generation of ^1^O_2_, in Figure 6b the reaction of ^1^O_2_ with **DMA**, in Figure 6c a representative example of monitorization of the reaction by UV-vis absorption spectroscopy and in Figure 6d the fitting of the data of a selected replica to the pseudo-first order kinetic model to obtain the corresponding observed kinetic constant (k_obs_). Note that, since **DMA** oxygenation is carried out using a heterogeneous photosensitizer, a notable variability in the calculated k_obs_ is obtained, and for this reason all the measurements were done by triplicate. The averaged values can be used to estimate the relative photoactivity of each material (Table 1).

**Figure 7:**
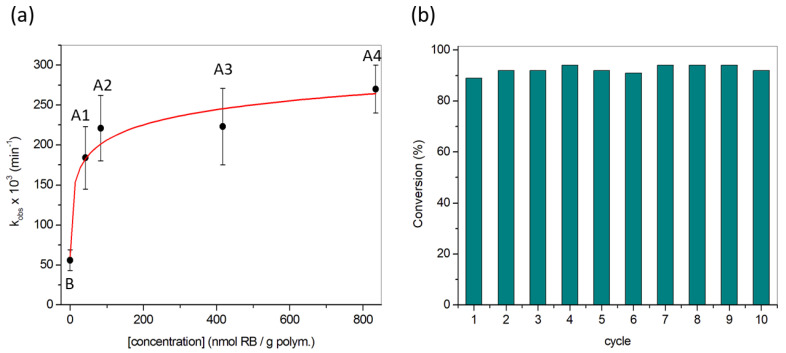
(**a**) Observed kinetic constants of the reaction between **DMA** and ^1^O_2_ generated by irradiated polymers **A1**–**A4**, **B**; (**b**) Conversion yield of the reaction between **DMA** and ^1^O_2_ generated by the same sample of irradiated polymer **A4** (see text for details) during 10 cycles.

Irradiations of polymers **A1**–**A4** in the presence of 10^−4^ M **DMA** in EtOH:H_2_O (1:1, v:v) were carried out using two white light lamps as energy sources (total irradiance 2 × 180 mW/cm^2^). The kinetic analysis of **DMA** absorption yielded constants in the range 184 to 270 × 10^−3^ min^−1^. K_obs_ values can be seen in Table 1 and Figure 7a. Self-sensitized oxidation of **DMA**, due to the residual absorption of this probe around 400 nm, must be considered since it yields a measurable value of k_obs_ (value for polymer **B**). The trend followed by the values of k_obs,_ as a function of **RB** concentration, is slightly ascending (significant statistical difference was found, *p ≤* 0.01, between control **B** and **A1**–**A4** films, but no within the **A1**–**A4** series). The curve is remarkably flat, which could be associated with several processes. One of the possibilities is that some degree of energy trapping would be taking place, even at such low concentrations of **RB** [67,68]. While this is out of the scope of the present study, further studies are required to clarify this hypothesis.

Another advantageous property of **A4** film is its reusability in multiple cycles of oxygenation [70]. Thus, in a first cycle, 50 mg of polymer **A4** was irradiated in the presence of 3 mL of 10^−4^ M **DMA** in EtOH:H_2_O (1:1, v:v) for 15 min. Then, the film was introduced in a freshly prepared solution of 10^−4^ M **DMA** in EtOH:H_2_O (1:1, v:v) and irradiated again for 15 min. This procedure was repeated up to 10 times (Figure 7b). After the tenth cycle, the colour of the material faded notably, but the polymer remained highly photoactive in terms of photooxygenation conversion (maintained at a constant value of ca. 90%). The total light dose received by the film in each irradiation cycle is 324 J/cm^2^, and after all the process, the total light dose received by the polymer is 3240 J/cm^2^.

### 3.3. Photodynamic Activity of RB@PHEMA Films against P. aeruginosa

**Figure 8:**
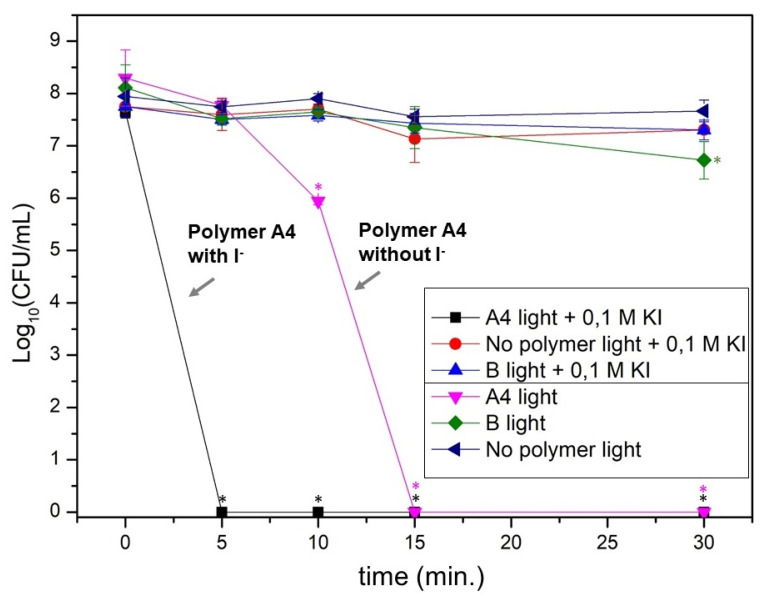
Photodynamic inactivation assays against *P. aeruginosa* with polymers **A4** or **B**, with and without iodide anion and with or without light (see legend for details). Asterisks (*) indicate significant differences with respect to value at time 0 min for each aPDI condition (*p* ≤ 0.01).

Planktonic suspensions of *P. aeruginosa* ATCC 27.853 (ca. 8 log_10_ CFU/mL initial load) were exposed to irradiation in the presence of **A4** film (fragments of 25 mg) with a source of white light for 5, 10, 15 and 30 min (light doses of 24, 48, 72 and 144 J/cm^2^, respectively). As can be seen in Figure 8, at a dose of 48 J/cm^2^ the population reduction was statistically significant (2 log_10_ CFU/mL) and at 72 J/cm^2^ the bacterial eradication was complete (8 log_10_ CFU/mL). Neither polymer **B** without **RB** nor irradiation without films yielded any reduction of the bacterial population. The eradication of *P. aeruginosa* using **RB** is very remarkable since it is well known that this pathogen is not appreciably inactivated by anionic **RB** in solution, although it is efficient for Gram-positive species [57]. The reason for this behavior can be ascribed to the fact that Gram-negative bacteria have a lipopolysaccharide (LPS) containing outer cell wall, making such bacteria much less susceptible to photosensitization [71]. Cationic photosensitizers (porphyrins, bodipys, etc) have been firmly established as good agents against this class of pathogens [71,72,73,74]. For this reason, the complete eradication of *P. aeruginosa* using an anionic photosensitizer like **RB** results notable. A similar effect has been described by the conjugation of non-cationic photosensitizers with cationic peptides, acting as potentiating molecules of the photodynamic activity (disrupting the wall and then allowing a more efficient attack of ^1^O_2_) [75]. 

The most remarkable aPDI effect was observed when irradiation of *P. aeruginosa* was carried out in the presence of potassium iodide (100 mM). As can be seen in Figure 8, the complete reduction of the bacterial population (8 log_10_ CFU/mL) occurs only with the minimum provided light dose of 24 J/cm^2^ (5 min. of irradiation) (*p* ≤ 0.01). Although the effect of iodide for irradiation with photosensitizers in solution has already been described (see Introduction), there are only two reports of this effect when using material-supported photosensitizers (one in the case of polystyrene fibers made by electrospinning [54] and the other one using nanoparticles of the same material [63]). The assays presented in this study describe the utility of **RB**-loaded PHEMA polymeric films to eradicate *P. aeruginosa* by irradiation as well as the antimicrobial activity enhancement by the addition of iodide anion.

The corresponding controls in the dark were also carried out, yielding no killing of the pathogen (not shown). This observation implies that the presence of ammonium groups in the structure of the PHEMA films, introduced by the monomer ATAC, is not enough to impart a significant antimicrobial activity in the dark, as it has been described for other materials [5,6,7,8,9,10,11,12,13,76,77].

It has been reported that, in solution, killing activity enhancement caused by iodide anion is due to the generation of both (a) short-lived reactive iodine species and (b) stable and long-term acting microbiocidal species like iodine/triiodide (I_2_/I_3_^−^). The prevalence of one or other species depends largely on the concentration of I^-^, being favoured the stable species by higher concentrations of it. Additionally, the group of Durantini has demonstrated enhanced intersystem crossing (ISC) to the first excited triplet state (T_1_) of bodipy photosensitizers when iodide anion is present in the medium, which also could enhance the formation of I_3_^-^ (via oxidation of I^-^ by ^1^O_2_) [59]. The exact mechanism for the generation of all the aforementioned species is not fully understood and the reader is referred to the pertinent literature for more details [61].

**Figure 9:**
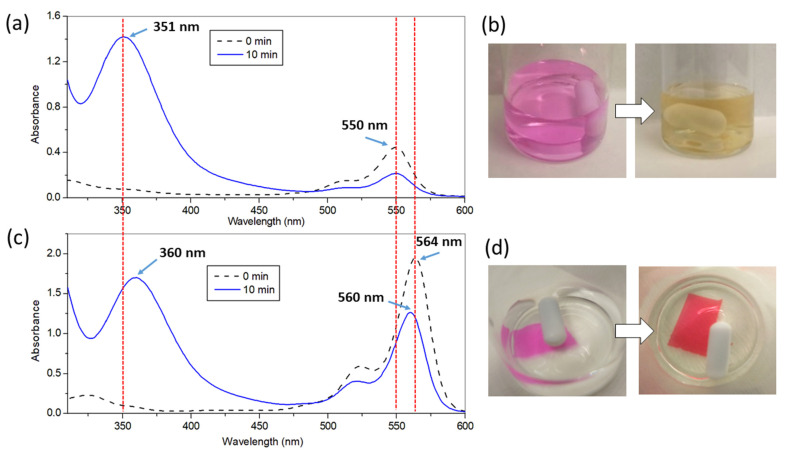
(**a**) UV-vis absorption spectra of a solution of **RB** (5 µM) and KI (0.1 M) before (dashed line) and after irradiation for 10 min (continuous line); (**b**) Visual aspect of the solution indicated in (**a**) at time 0 and after 48 min of irradiation; (**c**) UV-vis absorption spectra of an **A4** film immersed in KI solution (0.1 M) before (dashed line) and after irradiation for 10 min (continuous line); (**d**) Visual aspect of the solution and polymer indicated in (**c**) at time 0 and after 35 min of irradiation.

Our interest was to determine the actual formation of I_3_^−^ species since it is very easy to detect and follow due to their characteristic UV-vis absorption spectra, with peaks at 287 nm and 351 nm [54]. Thus, formation in solution was confirmed by irradiation of an aqueous solution of 100 mM I^−^ in the presence of **RB** 5 µM (Figure 9a). At 10 min of irradiation the formation of a band at 351 nm is a clear signature of I_3_^−^. In parallel to the rising of this band, there is progressive bleaching of the absorption of **RB** at 550 nm. Further irradiation up to 48 min led to the intensification of the I_3_^-^ band and complete bleaching of the **RB** absorption (illustrative picture in Figure 9b).

The same procedure was carried out using polymer **A4** as a source of ^1^O_2_ and interesting changes were noticed. First, I_3_^−^ formation was detected spectroscopically after 10 min of irradiation (Figure 9c) and by the naked eye after 35 min of irradiation (Figure 9d). The generated triiodide is entrapped electrostatically inside the film as evidenced by the fact that its absorption occurs shifted to 360 nm and by the colour change in the film (from pink to red). Considering the absorbance of the new band in the film, the extinction coefficient of I_3_^−^ (2.2·10^4^ M^−1^ cm^−1^) [78] and the thickness of the films, a rough calculation can estimate the concentration of this species inside the films at ca. 0.8 M. Such a high concentration of I_3_^−^ makes the irradiated polymers as truly permanent antibacterial materials. To check this hypothesis, the following assay was carried out: an **A4** film was irradiated in the presence of I^−^ for 15 min. and afterward the solution was spiked with *P. aeruginosa* (8 log_10_ CFU/mL). After 5 min of incubation in the dark, the bacterial killing was complete (data not shown). This long-term use, once finished the irradiation time, is coincident with the behaviour shown for photoactive polystyrene fibers [54,63].

Second, the irradiation of the film induced a small blue shift in the absorption of encapsulated **RB**, from 564 nm to 560 nm, probably indicating a change in the surrounding environment. Additionally, extended irradiation of the film up to 35 min yielded no additional bleaching of RB absorption (this is the reason why the film remains coloured in Figure 9d). All in all, the use of I^-^ in conjunction with entrapment of **RB** in the PHEMA matrix seems to provide a protective environment for the photosensitizer, hindering its complete photodegradation.

## 4. Conclusions

In summary, the non-covalent **RB** encapsulation in a series of PHEMA hydrogel films has led to photoactive materials generating ^1^O_2_, as demonstrated by the reactivity shown towards **DMA**. Polymer **A4**, with the highest **RB** load, possesses photobactericidal properties since it can eradicate a Gram-negative *P. aeruginosa* (planktonic state) population of 8 log_10_ CFU/mL at a total light dose of 72 J/cm^2^ (15 min. of white light exposure). Notably, this photodynamic action is highly enhanced by the addition of potassium iodide 100 mM, reducing the total light dose needed to 24 J/cm^2^ (5 min. irradiation time). The generation of bactericidal I_3_^-^ is demonstrated by the UV-absorption signature of this species. The photogenerated I_3_^-^ becomes trapped in the PHEMA matrix, at a high concentration (almost ca. 1 M), imparting to the polymer a long-lasting ability to kill bacteria.

## Figures and Tables

**Table 1:** Thermal properties of films **A1**–**A4** and **B** determined by TGA, and observed kinetic constant (average of three measurements) of the reaction of **DMA** with ^1^O_2_ generated by irradiation of the films.

Polymer	RB Loading (nmol RB/g Polymer)	*T*_5%_ (°C)	*T*_20%_ (°C)	*T*_max_ (°C)	k_obs_10^3^ (min^−1^)
A1	42	222.8	307.8	354.0	184 ± 39 ^a,b^
A2	83	227.9	311.9	352.9	221 ± 41 ^b^
A3	417	228.5	311.9	354.5	223 ± 48 ^b^
A4	835	225.9	311.4	359.7	270 ± 30 ^b^
B	0	219.8	312.4	366.8	56 ± 13 ^a^

^a,b^: *k_obs_* values sharing the same superscript letter are not significantly different from each other (one-way ANOVA, *p* ≤ 0.01).

## Supplementary Materials

The following are available online at https://www.mdpi.com/article/10.3390/polym13142227/s1, Figure S1: Experimental setup for the irradiations of polymers in the presence of singlet oxygen trap DMA, Figure S2: Experimental setup for the irradiations of polymers in the presence of bacteria.
