# Supplementary material for: Photodynamic Inactivation of Pseudomonas aeruginosa by PHEMA Films Loaded with Rose Bengal: Potentiation Effect of Potassium Iodide
Source: Polymers (Basel). 2021 Jul 6;13(14):2227. doi: 10.3390/polym13142227 (PMC8309320; doi:10.3390/polym13142227)
Supplement: Supplementary file 1 [file polymers-13-02227-s001.zip › polymers-1275698-supplementary.pdf]

Electronic Supporting Information

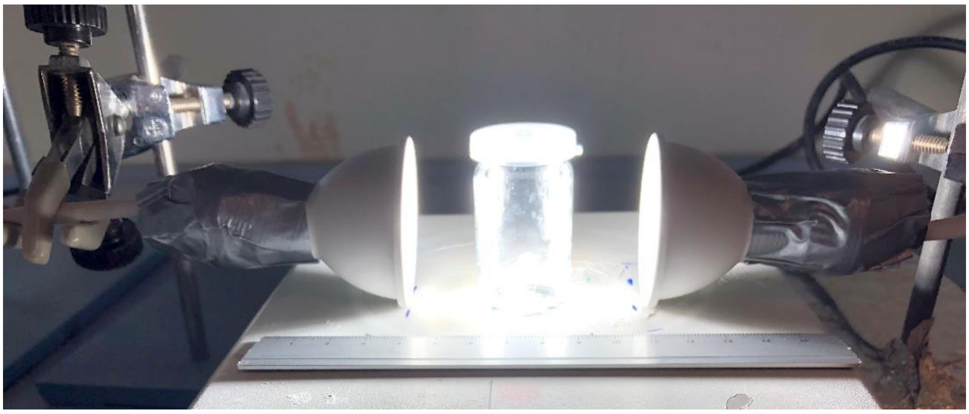

Figure S1. Experimental setup for the irradiations of polymers in the presence of singlet oxygen trap DMA.

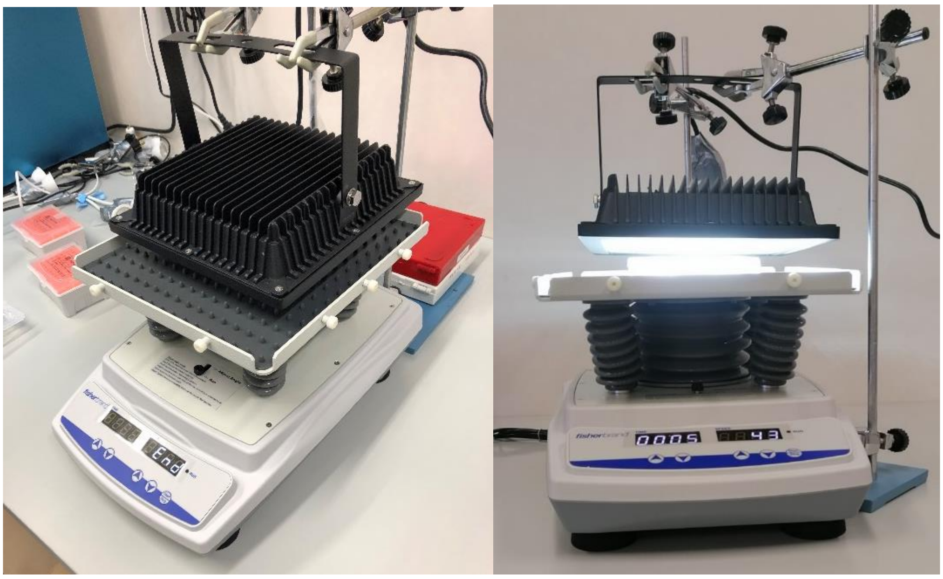

Figure S2. Experimental setup for the irradiations of polymers in the presence of bacteria.
